# Long-term outcomes following sacubitril/valsartan therapy for chronic HFrEF: an Italian real-world multicentre study

**DOI:** 10.1093/eschf/xvag082

**Published:** 2026-03-17

**Authors:** Giuseppe Dattilo, Roberto Licordari, Egidio Imbalzano, Antonio Cannata, Piergiuseppe Agostoni, Alberto Aimo, Francesco Barillà, Erberto Carluccio, Michele Ciccarelli, Gianluca Di Bella, Frank L Dini, Michele Emdin, Francesco Loria, Massimo Mapelli, Enrica Mariano, Francesco Paolo Niglio, Alberto Palazzuoli, Gianpaolo Palmieri, Simona Pavoncelli, Elisabetta Salvioni, Gianluigi Savarese, Michele Correale

**Affiliations:** Department of Biomedical and Dental Sciences and Morphofunctional Imaging, Section of Cardiology, University of Messina, Messina, Italy; Department of Biomedical and Dental Sciences and Morphofunctional Imaging, Section of Cardiology, University of Messina, Messina, Italy; Department of Clinical and Experimental Medicine, University of Messina, Via Consolare Valeria n.1, Messina 98125, Italy; British Heart Foundation Centre of Research Excellence, School of Cardiovascular Medicine, Faculty of Life Science, King's College London, London SW5 9RS, UK; Cardiology Department, King's College Hospital NHS Foundation Trust, London SE5 9NU, UK; Centro Cardiologico Monzino, IRCCs, Milan, Italy; Department of Clinical Sciences and Community Health, Section of Cardiology, University of Milan, Milan, Italy; Health Science Interdisciplinary Center, Scuola Superiore Sant’Anna, Pisa, Italy; Cardio-Thoracic Department, Fondazione Toscana Gabriele Monasterio, Pisa, Italy; U.O. Cardiologia, Fondazione Policlinico Tor Vergata, Roma, Italy; Cardiology and Cardiovascular Pathophysiology, S. Maria Della Misericordia Hospital, University of Perugia, Perugia, Italy; Department of Medicine, Surgery, and Dentistry, University of Salerno, Baronissi, Italy; Department of Clinical and Experimental Medicine, University of Messina, Via Consolare Valeria n.1, Messina 98125, Italy; Istituto Auxologico Italiano IRCCS, Centro Diagnostico e di Ricerca, Pioltello, Milano, Italy; Health Science Interdisciplinary Center, Scuola Superiore Sant’Anna, Pisa, Italy; Cardio-Thoracic Department, Fondazione Toscana Gabriele Monasterio, Pisa, Italy; Department of Medicine, Surgery, and Dentistry, University of Salerno, Baronissi, Italy; Centro Cardiologico Monzino, IRCCs, Milan, Italy; Department of Clinical Sciences and Community Health, Section of Cardiology, University of Milan, Milan, Italy; U.O. Cardiologia, Fondazione Policlinico Tor Vergata, Roma, Italy; Department of Medical and Surgical Sciences, University of Foggia, Foggia, Italy; Cardiovascular Diseases Unit Cardio-Thoracic and Vascular Department, S. Maria Alle Scotte Hospital, University of Siena, Siena, Italy; Department of Medical and Surgical Sciences, University of Foggia, Foggia, Italy; Cardiovascular Diseases Unit Cardio-Thoracic and Vascular Department, S. Maria Alle Scotte Hospital, University of Siena, Siena, Italy; Centro Cardiologico Monzino, IRCCs, Milan, Italy; Division of Cardiology, Department of Medicine, Karolinska Institutet, Stockholm, Sweden; Heart, Vascular and Neuro Theme, Karolinska University Hospital, Stockholm, Sweden; Cardiothoracic Department, Policlinico Riuniti University Hospital, Foggia, Italy

**Keywords:** Sacubitril/valsartan, Heart failure with reduced ejection fraction, Real-world evidence, Cardiac remodelling, Global longitudinal strain, NT-proBNP

## Abstract

**Background and Aims:**

Long-term real-world effects of sacubitril/valsartan (S/V) and the impact of S/V dose reduction or discontinuation are less defined. We assessed longitudinal changes after S/V initiation and the association of dose changes with major adverse cardiovascular events (MACE).

**Methods:**

Multicentre retrospective study of 592 HFrEF outpatients starting S/V (83% men; age 68 ± 10 years; LVEF 32 ± 7%). NT-proBNP, Kansas City Cardiomyopathy Questionnaire (KCCQ) and echocardiography were collected at baseline, 12 months, and last follow-up. MACE was analysed with Kaplan–Meier and Cox models.

**Results:**

NT-proBNP decreased from 1000 (494–2333) to 751 (304–1726) and 735 (215–1980) pg/ml (*P* < .001). KCCQ improved from 53 ± 15 to 62 ± 14 and 66 ± 15 (*P* < .001). LVEF increased from 32 ± 7 to 36 ± 8 and 37 ± 9% (*P* < .001) and GLS improved from −10.8 ± 3.2 to −12.3 ± 3.1 and −14.0 ± 2.9% (*P* < .001). During a median follow-up of 3.72 years, 225 patients (38%) experienced MACE (36 deaths; 134 HF hospitalizations). MACE incidence was higher in patients with S/V discontinuation and with dose reduction (log-rank *P* = .013 and *P* = .014). In multivariable Cox analysis, S/V discontinuation [hazard ratio (HR) 1.52, 95% confidence interval (CI) 1.28–1.97; *P* = .040], change in GLS (HR 0.81, 95% CI 0.67–0.98; *P* = .028) and change in KCCQ (HR 0.95, 95% CI 0.92–0.98; *P* = .001) were independently associated with MACE.

**Conclusions:**

S/V initiation was associated with sustained improvements in NT-proBNP, quality of life, and cardiac remodelling. S/V discontinuation or dose reduction identified patients at higher MACE risk.

## Graphical Abstract

**Graphical Abstract:**
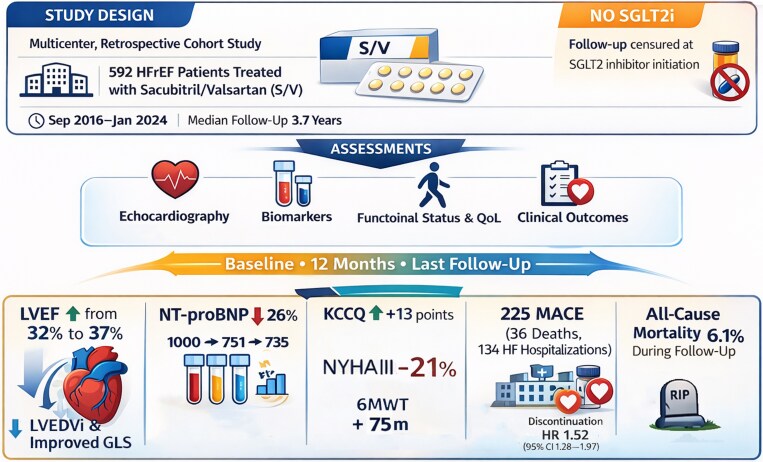

## Introduction

More than 64 million people worldwide suffer from heart failure (HF), and this clinical syndrome is considered to be a true pandemic.^1^ The prevalence of HF has increased by approximately 23% in the last 10 years due to the ageing of the population, with an incidence that varies with the age of the patients.^2–5^ Investigations of HFrEF, based on large registries, report 1-year mortality rates of 20% in patients recently hospitalized for HF and 6% in outpatients.^6^

The most recent guidelines of the European Society of Cardiology (ESC) for the pharmacological treatment of HFrEF recommend a simultaneous start of the (as first-line therapy) ‘four pillars”: renin-angiotensin system inhibitors (RASi) (preferably with an angiotensin receptor-neprilysin inhibitor (ARNi), or alternatively an ACE inhibitor/ARB when ARNi is not indicated or not tolerated), beta-blockers, mineralocorticoid receptor antagonists, and sodium-glucose cotransporter 2 inhibitors (SGLT2i).^7–9^

More data have emerged to support early and rapid initiation and titration of the ‘four pillars’ of medical therapy in HF to optimize the reduction in hospitalizations and mortality. More than one drug can be started and/or titrated at the same time. In some cases, a combination of the 4 drug classes can be started simultaneously at low doses according to guideline-based optimal medical therapy (GDMT) and more than one titration can be performed at a time.^10,11^ However, GDMT remains an unmet need in clinical practice.^12,13^ The publication of the Paradigm-HF study demonstrated that sacubitril/valsartan (S/V) (LCZ696) significantly reduced HF hospitalization and cardiovascular mortality in HFrEF patients, regardless of HF aetiology.^14,15^ S/V was the first of the four pillars to be introduced in patients with HFrEF according to the 2016 ESC guidelines^16^; In this real-world multicentre study, we aimed to evaluate the long-term effectiveness of S/V therapy in patients with HFrEF. Specifically, we focused on changes in echocardiographic parameters, NYHA class, KCCQ scores, and 6MWT scores during the first year of treatment. We then assessed whether these improvements plateaued, continued to improve, or reversed during the subsequent years of follow-up. This evaluation was conducted prior to the introduction of SGLT2i therapy.^17–28^

## Methods

### Study design and participants

This multicentre retrospective study collected patient data from 13 Italian outpatient centres for HF. All patients diagnosed with HFrEF who were introduced to S/V therapy according to the indications of the 2016 ESC Guidelines were enrolled. The enrolment period was from September 2016 to January 2024. Follow-up was censored at the time of SGLT2i initiation for HF indication. Patients with limitations to physical autonomy were excluded from enrolment. Patients who underwent cardiac surgery, advanced kidney disease, pacemaker (PM) implants for cardiac resynchronization therapy, orthopaedic surgery, or major general surgery during the follow-up period were excluded from the study (Supplementary Figure S1). A comparison between two consecutive follow-up periods was conducted only for patients with available information on the variables of interest: (i) at 12 months since S/V introduction and (ii) from the 12 months up to SGLT2i introduction. The main objective was to evaluate the effects of S/V in a real-world population over an extended follow-up by a multiparametric approach: (i) echocardiographic assessment of LV dimension, systolic and diastolic function; (iii) functional capacity by the 6-min walking test (6MWT); (iii) evaluation of the perceived quality of life by the administration of the Kansas City Cardiomyopathy Questionnaire (KCCQ) and monitoring of the trend of biomarkers (renal function, BNP, NTproBNP); and (iv) incidence of major adverse cardiovascular events (MACE: HF rehospitalization, all-cause death, and new-onset of atrial fibrillation).^29–33^ The study adhered to the principles of the Declaration of Helsinki and was submitted for review to the ethics committee of each participating centre.

### Statistical analysis

The Kolmogorov–Smirnov test was performed to assess the normality of the data distribution. Variables with a normal distribution are presented as mean ± standard deviation (SD), while non-normally distributed variables are shown as median and interquartile range (IQR, 25th–75th percentile). For variables with a normal distribution, repeated-measures analysis of variance with Greenhouse–Geisser correction was used to compare clinical, echocardiographic, and laboratory parameters at baseline, 12 months follow-up, and the last follow-up visit. Post-hoc pairwise comparisons were adjusted for multiple comparisons using Bonferroni correction. For non-normally distributed variables, the Friedman test was applied to analyse changes over time, with Durbin-Conover tests used for multiple comparisons. For the composite endpoint (MACE), a time-to-first-event approach was adopted; in patients experiencing multiple component events during follow-up, only the first event in chronological order was considered for the MACE analysis. Survival analysis was conducted using Kaplan–Meier curves for each individual endpoint and the composite endpoint. Differences between groups were assessed using the log-rank test. Univariate Cox regression analyses were performed for the combined endpoint and individual endpoints. In multivariate models, clinically relevant and/or statistically significant variables in the univariate analysis were included, ensuring that multicollinearity was avoided. Missing data were summarized and reported. Analyses were performed on an available-case (complete-case for each specific model) basis without imputation; denominators vary accordingly and are shown in tables. A *P*-value < .05 was considered statistically significant. All statistical analysis was performed using RStudio (version 2022.07.2; Integrated Development Environment for R. Boston, MA, USA).

## Results

The study population consisted of 592 patients, 83% male, with a mean age of 68 ± 10 years. The baseline characteristics of the population are presented in *Table 1*. The mean LVEF at baseline was 32% ± 7. In 352 patients (60%), the aetiology of HF was ischaemic. 164 patients (28%) had received CRT, and 303 patients (51%) had an ICD.

**Table 1:** Baseline characteristics of the study population

Characteristic	*N* = 592
Age (years)	68 (10)
Gender	
F	99 (17%)
M	493 (83%)
Weight (kg)	80 (15)
Height (cm)	170 (8)
BMI (kg/m^2^)	27.5 (4.5)
BSA (m^2^)	1.94 (0.21)
Implantable cardioverter device	303 (51%)
Cardiac resynchronization therapy	164 (28%)
Diabetes mellitus	217 (37%)
Hypertension	467 (79%)
History of atrial fibrillation	204 (34%)
Non-ischaemic dilated cardiomyopathy	222 (38%)
Ischaemic heart disease	352 (60%)

### Changes of clinical, laboratory, and echocardiographic parameters during the follow-up

**Figure 1:**
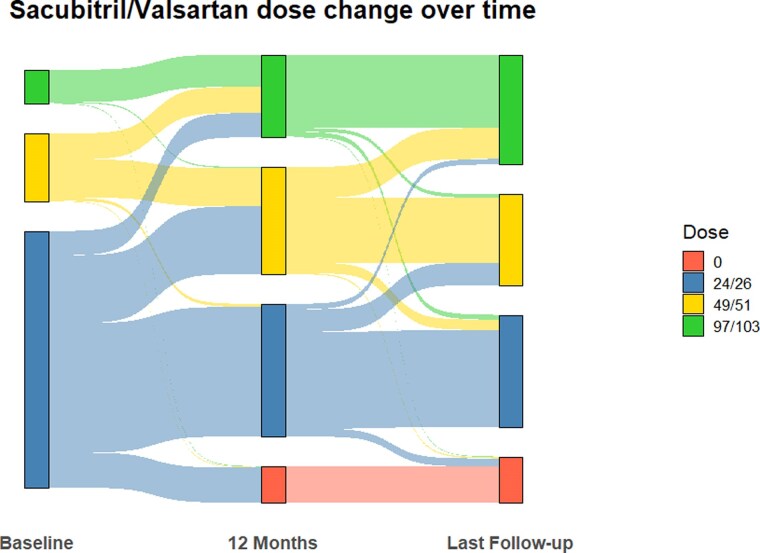
Sankey diagram illustrating the changes in sacubitril/valsartan dosing over time

At baseline, all patients were naïve to the use of ARNI. All patients were prescribed S/V during follow-up, with up-titration, as shown in *Figure 1* and *Table 2*. Specifically, 181 patients (31%) were treated with S/V at 97/103 mg by the last follow-up, compared with 136 patients (23%) at the 12-month assessment. Additionally, 60 patients (10%) discontinued the drug by 12 months, while 76 patients (13%) had stopped S/V by the last follow-up. Common reasons for dose reduction or discontinuation included symptomatic hypotension, worsening renal function, and patient intolerance (e.g. dizziness, fatigue). Supplementary Table S1 reports differences in baseline characteristics between patients who discontinued or reduced S/V dosage during follow-up and patients who maintained or up-titrated S/V dosage. Briefly, patients who reduced or discontinued S/V therapy were generally older, had lower baseline blood pressure, and higher baseline NTproBNP levels, suggesting a more fragile clinical profile.

**Table 2:** Key clinical and laboratory parameters and their changes during follow-up

Characteristic	Baseline	12 months	Last follow-up	*P*-value
SBP (mmHg)	126 (16)	120 (16)	119 (15)	<.001
DBP (mmHg)	73 (10)	70 (10)	70 (10)	<.001
Creatinine (mg/dl)	1.15 (0.37)	1.18 (0.43)	1.40 (0.87)	<.001
Na (mEq/L)	139.58 (3.00)	139.98 (3.03)	139.83 (2.99)	.013
K (mEq/L)	4.28 (0.47)	4.37 (0.41)	4.48 (0.49)	<0.001
Hb (g/dl)	13.39 (1.64)	13.33 (1.75)	13.55 (1.97)	0.2
NTproBNP (pg/ml)	1000 (494–2333)	751 (304–1726)	735 (215–1980)	<0.001
BNP (pg/ml)	235 (106–510)	176 (120–245)	165 (143–322)	0.046
NYHA Class				<0.001
1	33 (5.6%)	108 (19%)	163 (29%)	
2	347 (59%)	371 (65%)	317 (56%)	
3	210 (35%)	90 (16%)	82 (14%)	
4	2 (0.3%)	2 (0.4%)	4 (0.7%)	
6MWT (m)	283 (78)	338 (77)	358 (78)	<0.001
KCCQ Score	53 (15)	62 (14)	66 (15)	<0.001
S/V Dose				<0.001
0	0 (0%)	60 (10%)	76 (13%)	
24/26	424 (72%)	219 (37%)	185 (31%)	
49/51	112 (19%)	177 (30%)	150 (25%)	
97/103	56 (9.5%)	136 (23%)	181 (31%)	
Beta-blockers	443/487 (91%)	439/485 (91%)	430/476 (90%)	>0.9
MRA	336/483 (70%)	350/471 (74%)	354/458 (77%)	0.025
Ivabradine	45/487 (9.2%)	49/471 (10%)	46/456 (10%)	>0.9
Digoxin	52/487 (11%)	49/471 (10%)	54/458 (12%)	0.8
SGLT2i^a^	40/217 (18.4%)	44/217 (20.3%)	52/217 (24%)	0.353

6MWT, 6-min walking test; BNP, B-type natriuretic peptide; DBP, diastolic blood pressure; Hb, haemoglobin; K, potassium; KCCQ, Kansas City Cardiomyopathy Questionnaire Score; MRA, mineral receptor antagonist; Na, sodium; NTproBNP, N-terminal pro B-type natriuretic peptide; NYHA, New York Heart Association Functional Class; S/V, sacubitril/valsartan; SBP, systolic blood pressure.

^a^SGLT2i reported among patients with diabetes.

Table 32 shows the patients’ key clinical and laboratory characteristics and changes across the three-time points. SBP and DBP showed a significant decline during the first 12 months, followed by a slight decrease until the last evaluation (*P* < .001). NTproBNP levels decreased significantly across the three-time points (*P* < .001). Among the clinical parameters, the 6MWT and KCCQ scores showed significant improvements (*P* < .001 for both), while the NYHA functional class significantly declined during the follow-up (*P* < .001).

**Figure 2:**
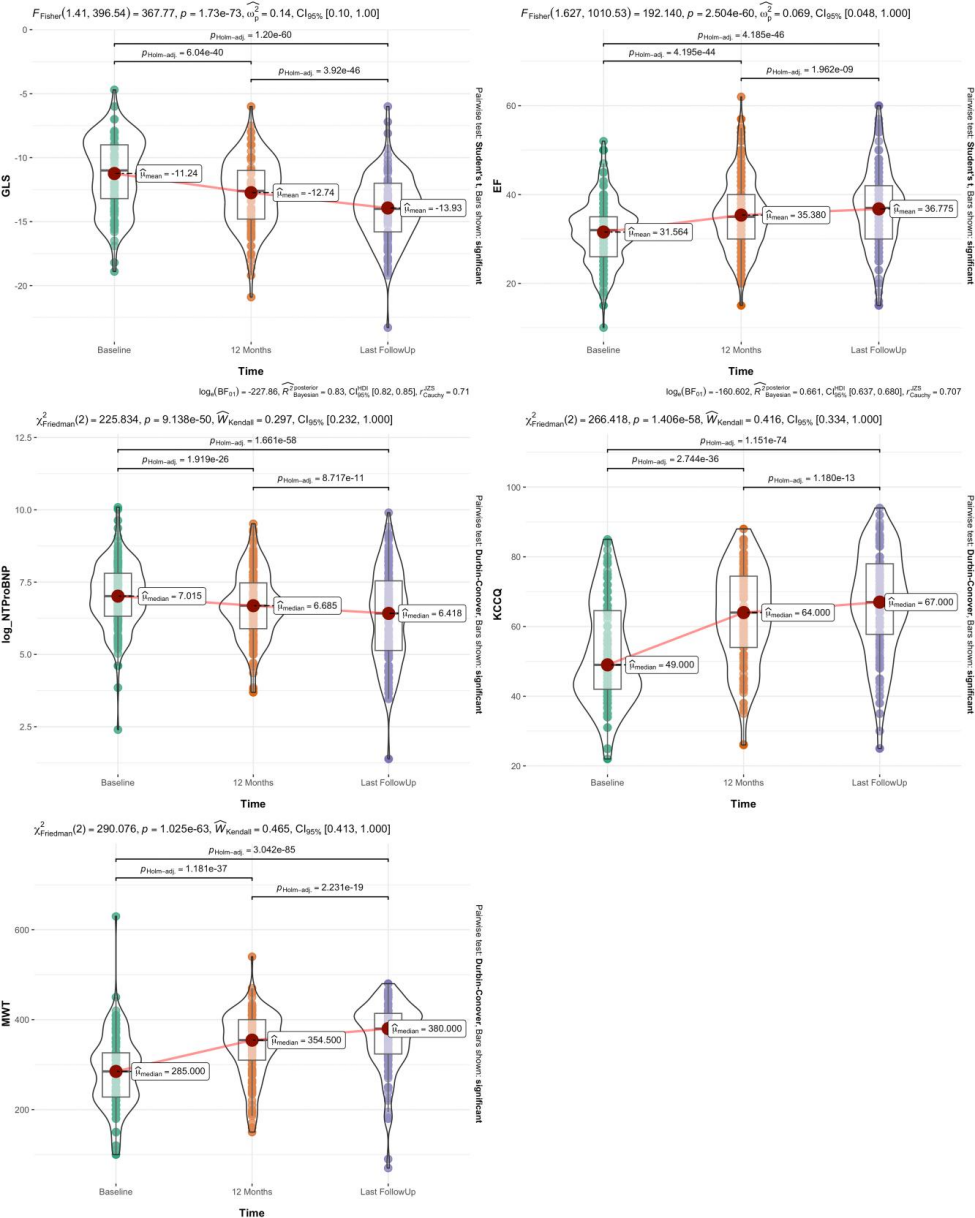
Box/violin plots illustrating the trends in global longitudinal strain (GLS, panel *A*), left ventricular ejection fraction (LVEF, panel *B*), NTproBNP (panel *C*), Kansas City Cardiomyopathy Questionnaire (KCCQ) score (panel *D*), and 6-min walk test (6MWT, panel *E*) over time

*Table 3* reports the changes over time in the key echocardiographic parameters. Left ventricle end-diastolic volume (LV EDV) (*P* < .001), left ventricle end-systolic volume (LV ESV) (*P* < .001), the ratio of LV early diastolic mitral inflow to early diastolic mitral annular velocity (E/e’ ratio) (*P* < .001), pulmonary artery systolic pressure (sPAP) (*P* = .006), and global longitudinal strain (GLS) (*P* < .001) all showed an improvement during follow-up, whereas LVEF (*P* < .001), TAPSE (*P* = .009), and tissue doppler imaging parameters significantly increased. These results are further detailed in *Figure 2*.

**Table 3:** Key echocardiographic parameters and their changes during follow-up

Characteristic	Baseline	12 months	Last follow-up	*P*-value
LVEDV (ml)	175 (58)	166 (59)	157 (61)	<.001
LVEDVi (ml/m^2^)	92 (31)	87 (30)	82 (31)	<.001
LVESV (ml)	122 (51)	110 (48)	102 (52)	<.001
LVESVi (ml/m^2^)	64 (27)	57 (25)	54 (26)	<.001
LVEF (%)	32 (7)	36 (8)	37 (9)	<.001
E/e’	13.5 (6.0)	12.0 (4.8)	11.3 (4.6)	<.001
S’ mean (cm/s)	6.71 (2.46)	7.02 (2.52)	7.40 (3.05)	.036
TAPSE (mm)	19.3 (4.1)	19.6 (3.9)	20.1 (4.3)	.009
S’ tricuspid (cm/s)	10.38 (2.29)	10.98 (2.51)	11.34 (2.56)	<.001
sPAP (mmHg)	34 (10)	33 (10)	32 (9)	.006
LAV (ml)	67 (31)	66 (31)	63 (30)	.090
LAVi (ml/m^2^)	35 (16)	34 (16)	32 (15)	.059
GLS (%)	−10.8 (3.2)	−12.3 (3.1)	−14.0 (2.9)	<.001

E/e’, E/e’ ratio; GLS, global longitudinal strain; LAV, left atrial volume; LVEDV, left ventricle end-diastolic volume; LVESV, left ventricle end-systolic volume; LVEF, left ventricle ejection fraction; S'mean, mean mitral S’; S'tricuspid, Tricuspid S’; sPAP, systolic pulmonary artery pressure; TAPSE, tricuspid annular plane systolic excursion.

As shown in *Figure 2*, GLS (panel *A*) and the logarithmic transformation of NTproBNP values (panel *C*) exhibited a significant improvement across all time points, including between the 12-month evaluation and the last follow-up. Similarly, LV EF (panel *B*), KCCQ score (panel *D*), and 6MWT distance (panel *E*) showed a significant improvement throughout the three evaluations, including the interval between 12 months and the last follow-up.

**Figure 3:**
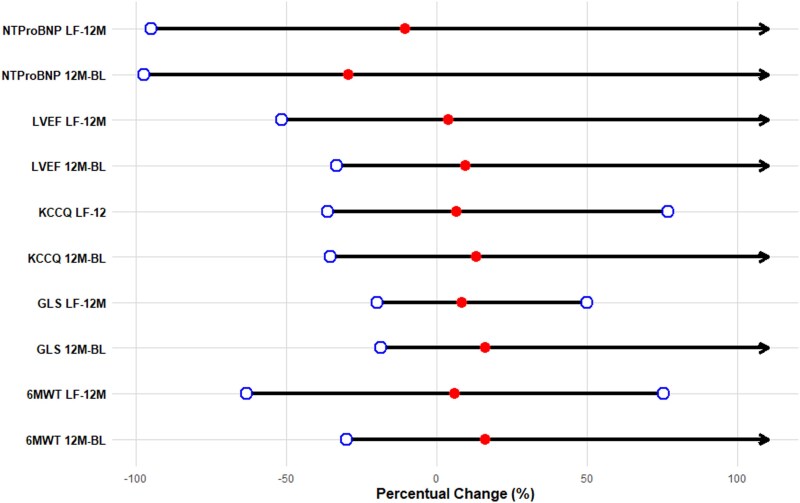
Forest plot illustrating the percentage changes in NTproBNP, left ventricular ejection fraction (LVEF), Kansas City Cardiomyopathy Questionnaire (KCCQ) score, global longitudinal strain (GLS), and 6-min walk test (6MWT) across two time intervals: from baseline (BL) to 12 months (12 M) and from 12 months (12 M) to the last follow-up (LF). The plot visualizes the medians and ranges of percentage changes for each parameter, with arrows indicating extreme values

The median percentage changes and interquartile ranges for the analysed parameters showed significant variations between baseline and 12 months and between 12 months and the last follow-up. Specifically, NTproBNP levels exhibited a median reduction of 29% (−50, −5) between baseline and 12 months, followed by a further median decrease of 11% (−45, 5) between 12 months and the last follow-up. LVEF improved with a median increase of 9% (0, 22) at 12 months compared with baseline and a subsequent median rise of 4% (−5, 11) from 12 months to the last follow-up. Similarly, GLS showed a median change of 16% (5, 25) between baseline and 12 months, followed by an additional 8% (3, 17) between 12 months and the last evaluation. The 6MWT distance and KCCQ score demonstrated significant improvements throughout the follow-up, with further details on these changes in the forest plot (*Figure 3*). Comparisons of these parameters between subgroups stratified by S/V therapy discontinuation or dosage are reported in Supplementary Table S2.

### Clinical outcomes

As shown in *Table 4*, during a median clinical follow-up of 3.72 years (IQR 1.9–4), 225 patients experienced MACE. In particular, 36 patients (6.1%) died, HF rehospitalization occurred in 134 patients (23%), and new-onset AF occurred in 133/388 patients without prior AF (34%); overall 133/592 (22%).

**Table 4:** Clinical events recorded during follow-up

Characteristic	*N* = 592
All-cause death	36 (6.1%)
HF hospitalization	134 (23%)
New onset atrial fibrillation	133 (22%)^a^
MACE (time-to-first event)	225 (38%)
Follow-up (days)	1339 (686–1460)

^a^34% among patients at risk.

*Table 5* presents the univariate and multivariate Cox regression analyses for key clinical, echocardiographic, and laboratory parameters about the combined endpoint of MACE. In the multivariate analysis, independent predictors of MACE included the change in GLS (calculated as GLS at baseline minus GLS at 12 months), the change in KCCQ score (calculated as KCCQ score at 12 months minus baseline score), the logarithmic change in NTproBNP during the first 12 months, and discontinuation of S/V during follow-up. Specifically, a 1-unit increase in (baseline–12 M) GLS was associated with an approximate 19% reduction in the risk of MACE [hazard ratio (HR) = .81, 95% confidence interval (CI): 0.67–0.98, *P* = .028], while an increase in KCCQ score was linked to a 5% reduction in risk (HR =0.95, 95% CI: 0.92–0.98, *P* = .001). Conversely, discontinuation of S/V was associated with a higher risk of MACE (HR = 1.52, 95% CI: 1.28–1.97, *P* = .040).

**Table 5:** Univariate and multivariate cox regression analysis for Major adverse cardiovascular events (MACE)

Characteristic	Univariate			Multivariate		
	HR	95% CI	*P*-value	HR	95% CI	*P*-value
Age (years)	1.03	1.01, 1.04	<.001	1.03	1.00, 1.06	.058
Baseline GLS (%)	0.90	0.85, 0.95	<.001			
**Change in GLS**	0.66	0.58, 0.75	<.001	0.81	0.67, 0.98	.028
History of AF	2.90	2.23, 3.77	<.001			
Baseline SBP (mmHg)	0.99	0.99, 1.00	.15			
Baseline LVEF (%)	1.00	0.98, 1.02	.78			
Change in LVEF	0.98	0.96, 1.00	.11	1.02	0.97, 1.06	.45
Baseline 6MWT (m)	1.00	1.00, 1.00	.085			
Baseline KCCQ Score	0.97	0.96, 0.98	<.001			
**Change in KCCQ Score**	0.93	0.92, 0.95	<.001	0.95	0.92, 0.98	.001
Baseline TAPSE (mm)	0.99	0.96, 1.02	.42			
Baseline LAV (ml)	1.00	0.99, 1.00	.40			
Baseline Creatinine (mg/dl)	1.43	0.95, 2.18	.090			
Log baseline NTproBNP (pg/ml)	1.04	0.92, 1.18	.50			
Log change in NTProBNP	1.52	1.22, 1.89	<.001	1.36	0.96, 1.94	.087
Baseline beta-blocker therapy	0.58	0.37, 0.90	.014	0.77	0.44, 1.36	.37
Baseline MRA therapy	1.12	0.83, 1.52	.46			
**S/V discontinuation**	1.60	1.10, 2.33	.014	1.52	1.28, 1.97	.040

6MWT, 6-min walking test; GLS, global longitudinal strain; KCCQ, Kansas City Cardiomyopathy Questionnaire Score; LAV, left atrial volume; LVEF, left ventricle ejection fraction; NTproBNP, N-terminal pro B-type natriuretic peptide; S/V, sacubitril/valsartan; TAPSE, tricuspid annular plane systolic excursion.

Given the baseline clinical differences between patients who discontinued S/V and those who maintained therapy, we performed a sensitivity multivariable Cox analysis including established baseline severity markers (age, history of atrial fibrillation, baseline systolic blood pressure, baseline log-transformed NT-proBNP, and baseline NYHA class). In this model, S/V discontinuation remained independently associated with MACE (adjusted HR 1.60, 95% CI 1.04–2.48; *P* = .034) (Supplementary Table S3).

**Figure 4:**
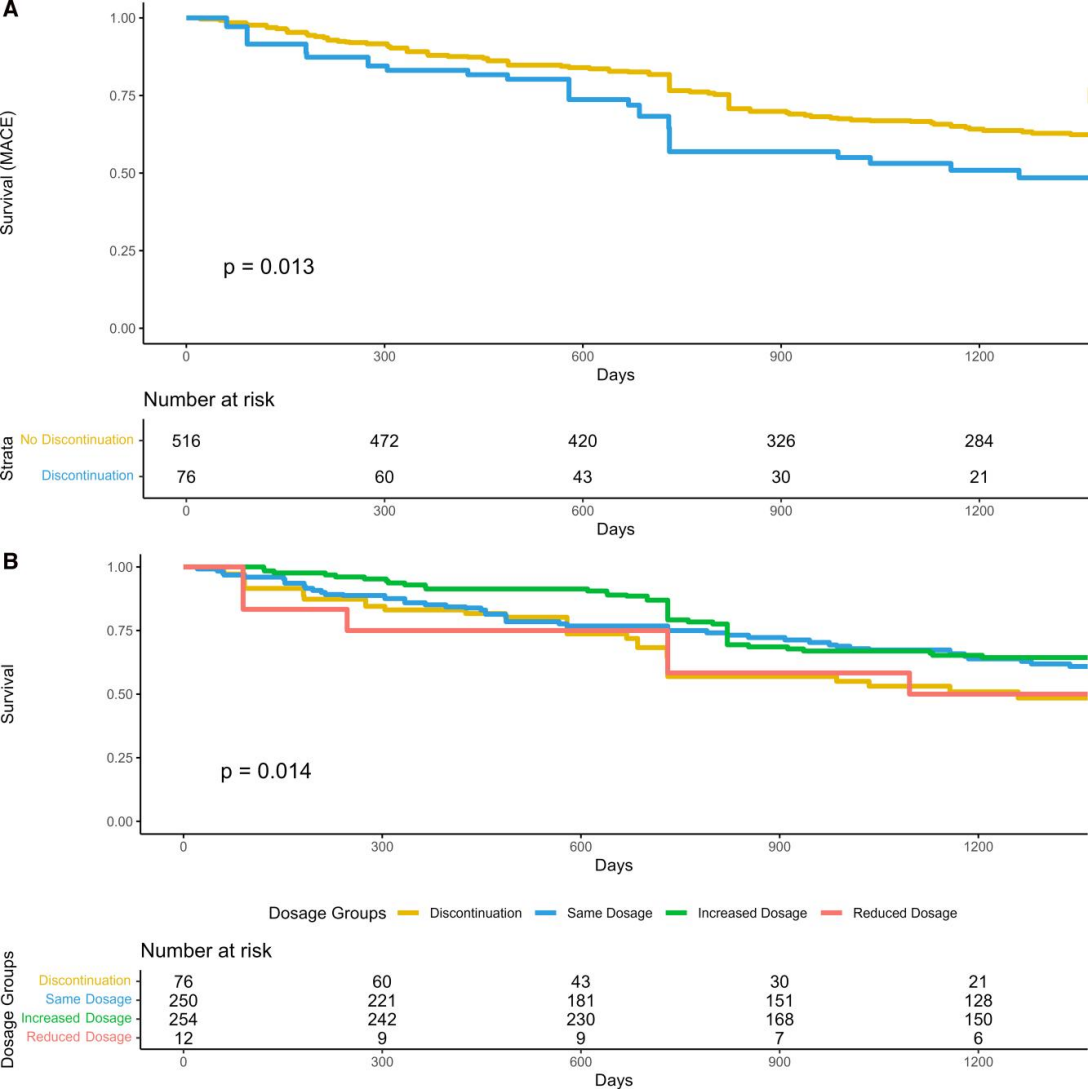
Kaplan–Meier analysis for major adverse cardiovascular events (MACE). Panel A shows the survival curves for patients who discontinued vs. those who continued sacubitril/valsartan therapy. Panel B illustrates the survival curves based on Sacubitril/Valsartan dose adjustments during follow-up, categorizing patients into those who increased, maintained, reduced the dosage, or stopped the drug. Kaplan–Meier curves are displayed up to 3.5 years because the number of patients at risk became small thereafter

Cox regressions identifying predictors of all-cause mortality, HF rehospitalization, and atrial fibrillation are reported in Supplementary Tables S4–S6. Finally, *Figure 4* displays the Kaplan–Meier survival curves for MACE. Panel A compares patients who discontinued S/V versus those who continued therapy, while Panel B presents the survival curves stratified by dose adjustment during follow-up. Patients who discontinued S/V were at a significantly higher risk of MACE during follow-up (log-rank test *P* = .013). *Figure 4* Panel B provides an exploratory, descriptive comparison across dose-adjustment categories; interpretation is limited by unbalanced subgroup sizes and the decreasing number at risk over time, but it shows a similar trend in patients who experienced dose reduction during follow-up (log-rank test *P* = .014). Particularly, significant difference emerged between patients who discontinued therapy and those who experienced dose up-titration (*adjusted P* = .0074).

The amount of missing data across key variables and time points is summarized in Supplementary Table S7.

## Discussion

HF is a chronic condition characterized by a progressive worsening, often requiring hospitalization and intravenous diuretic treatment, associated with a further deterioration of prognosis and quality of life.^34–37^ This is the first study reporting an extended and complete clinical and echocardiographic follow-up (median clinical follow-up of 3.72 years) for outpatients treated with S/V therapy. Our multicentre, retrospective, real-world study analysed data from medical records of HF patients collected at thirteen specialized centres across Italy. It aimed to elucidate whether the improvements achieved in the first year of S/V use^19–28^ were maintained in subsequent years. A total of 592 patients with similar demographic and clinical characteristics were evaluated. We observed that the improvements obtained in the first year of S/V administration in patients with HFrEF, in terms of GLS, EF, 6MWT, KCCQ, and NTproBNP, continued to improve in the following years, even to a lesser extent than in the first year. Indeed, as indicated in *Figure 3*, the prognosis of HFrEF can significantly improve since the amelioration of cardiac performance, resistance to physical effort, the improvement of the patient's perceived quality of life, and the reduction of NTproBNP counteract the recurrence of HF exacerbation and hospitalization.

Notably, improvements in functional and biological surrogate markers are not invariably required to achieve reductions in clinical events. In interventional strategies such as transcatheter edge-to-edge repair (TEER) for secondary mitral regurgitation, randomized trials have reported meaningful reductions in HF hospitalizations (and, in selected populations, mortality), while changes in conventional functional metrics or biomarkers have been modest, variable, or not consistently reported across studies.^38^ This apparent dissociation supports the concept that different therapies may improve outcomes through distinct pathways: TEER primarily reduces regurgitant volume and left-sided filling pressures (thereby lowering decompensation risk), whereas ARNI therapy is expected to exert disease-modifying effects through neurohormonal modulation and reverse remodelling, which are more directly reflected by changes in LVEF/GLS, natriuretic peptides and patient-reported outcomes.^39^

The data from a prospective observational registry of the Heart Failure Association of the European Society of Cardiology, published in 2016,^6^ to which 211 cardiac centres in 21 European countries contributed, between May 2011 and April 2013 and data on 12 440 patients, reports 1-year mortality rates of 6% in outpatients with HFrEF. In our cohort, all-cause mortality was 6.1% over a median follow-up of 3.72 years. This rate appears lower than that reported in other HFrEF cohorts, including randomized trial populations.^14^ It is possible that these results reflect the specific characteristics of our study population: ambulatory outpatients managed in specialized HF centres, eligibility for ARNI initiation with preserved physical autonomy, and a high prevalence of device therapy. On the other hand, these findings suggest that a favourable prognostic profile can be observed in ambulatory HFrEF patients managed in specialized HF clinics and treated with contemporary GDMT, including S/V. The problem that emerges most from the latest scientific evidence is that the implementation status of GDMT still needs to be improved worldwide, which could lead to a reduction in terms of mortality.^12,40^

In our study population, medical therapy influenced by the 2016 guidelines was associated with long-term lower rates of mortality, HF rehospitalization and MACE, with improved survival in patients who did not discontinue therapy (*Figure 4*). Our findings support an association between the ability to up-titrate S/V and better outcomes. Our survival curves diverge early and show better prognosis for patients able to increase the dose of S/V during an extended follow-up. Patients who discontinued S/V are at a significantly higher risk of MACE during an extended follow-up. A decrease in beta-blocker therapy was observed in the S/V discontinuation group (Supplementary Table S2). This finding should be interpreted cautiously due to the high event-rate in this group; certainly, it is consistent with a more vulnerable phenotype and reduced tolerability to multiple components of guideline-directed therapy rather than a drug-specific effect. However, a similar high risk of MACE is observed in patients who experienced dose reduction during an extended follow-up. Recently, data from a retrospective cohort study with extended follow-up (<6 years) support the long-term beneficial effects (risk of hospitalization for a cardiovascular reason and for HF) of S/V in older patients and in those experiencing the most severe symptoms.^41^

### Limitations

This is a retrospective, observational multicentre study and is therefore subject to selection bias, information bias (including missing or inconsistently recorded variables), and residual confounding. In particular, dose reduction or discontinuation of S/V is not randomly assigned and, in routine practice, often reflects reduced tolerability and/or a more advanced HF phenotype (confounding by indication/tolerability); accordingly, outcome associations should be interpreted as descriptive and hypothesis-generating rather than causal. Moreover, the absence of a concurrent control group (e.g. patients maintained on ACEi/ARB) precludes causal inference, and the observed improvements in LVEF, GLS, NT-proBNP, and KCCQ cannot be attributed exclusively to S/V, as concomitant optimization of guideline-directed therapy and comprehensive HF clinic care may have contributed. The comparison with historical registry data is descriptive and does not replace a concurrent control group.

Generalizability may also be limited by the outpatient, specialized-centre setting and selection criteria, and outcome ascertainment based on medical records may have led to incomplete capture of events occurring outside participating centres. Several clinically relevant variables were not available in a standardized manner across centres, including HF duration and the timing/number of prior decompensation episodes, prior ACEi/ARB exposure (agent and duration), loop diuretic use and dosing, and advanced HF management pathways (e.g. referral for heart transplantation, LVAD evaluation, palliative care, or multidisciplinary advanced HF discussion). In addition, all-cause and cardiovascular hospitalizations could not be reliably adjudicated; therefore, outcomes were restricted to death and HF hospitalization, while new-onset atrial fibrillation was included as a pragmatic marker of clinically relevant deterioration, which may limit comparability with other composite endpoints.

SGLT2 inhibitor exposure prescribed as glucose-lowering therapy was available and is reported; however, follow-up was defined up to initiation of SGLT2 inhibitors, so findings reflect S/V use in a pre–SGLT2i (or early SGLT2i) therapeutic context and may not fully generalize to contemporary practice where SGLT2i are routinely co-initiated as part of the four foundational therapies. Finally, longitudinal medication data were not uniformly available at all time points; therefore, medication proportions over time are descriptive and denominators may vary due to missing data and events (including death), and changes in background therapies (e.g. beta-blockers) should be interpreted cautiously.

## Conclusion

In this multicentre longitudinal retrospective study, the benefits of S/V in terms of echocardiographic parameters, NYHA functional class, 6MWT, KCCQ, and NTproBNP, occurred progressively throughout the follow-up period. These benefits were obtained mainly during the first year of administration and, although with a lower % change, continued in the following years.

## Supplementary Material

xvag082_Supplementary_Data

## Declarations

### Data Availability

No data were generated or analysed for this manuscript.

### Pre-registered Clinical Trial Number

None supplied.
