# Supplementary material for: Long-term outcomes following sacubitril/valsartan therapy for chronic HFrEF: an Italian real-world multicentre study
Source: ESC Heart Fail. 2026 Mar 17;13(2):xvag082. doi: 10.1093/eschf/xvag082 (PMC13122607; doi:10.1093/eschf/xvag082)

**Supplementary Table 1:** Differences in baseline features between patients who reduced dosage or discontinued S/V therapy versus patients who maintained or up-titrated S/V dosage.

| **Characteristic** | **S/V Reduced or Discontinued**   N = 113 | **S/V Maintained or Up-titrated** N = 479 | **p-value** |
| --- | --- | --- | --- |
| Age (years) | 72 (9) | 67 (10) | <0.001 |
| Gender |  |  | >0.9 |
| F | 18 (16%) | 81 (17%) |  |
| M | 95 (84%) | 398 (83%) |  |
| Weight (kg) | 77 (13) | 80 (16) | 0.2 |
| Height (cm) | 170 (7) | 170 (8) | 0.5 |
| ICD | 58 (51%) | 245 (51%) | >0.9 |
| CRT | 28 (25%) | 136 (28%) | 0.5 |
| Diabetes Mellitus | 46 (41%) | 171 (36%) | 0.4 |
| Hypertension | 98 (88%) | 369 (78%) | 0.026 |
| History of Atrial Fibrillation | 56 (50%) | 148 (31%) | <0.001 |
| Non-ischaemic Dilated Cardiomyopathy | 30 (27%) | 192 (40%) | 0.011 |
| Ischaemic Heart Disease | 78 (70%) | 274 (58%) | 0.025 |
| Baseline Systolic Blood Pressure (mmHg) | 123 (14) | 126 (16) | 0.011 |
| Baseline NTproBNP (pg/mL) | 1,500 (800 - 3,215) | 854 (450 - 1,900) | <0.001 |

| **Supplementary Table 2:** Comparison of different stratified parameters between different subgroups | | | | | |
| --- | --- | --- | --- | --- | --- |
| **Characteristic** | **Discontinuation**  N = 76 | **Same Dosage**  N = 250 | **Up-titration** N = 254 | **Down-titration** N = 12 | **p-value** |
| Age (years) | 71 (10) | 68 (10) | 66 (10) | 71 (9) | **<0.001** |
| Gender |  |  |  |  | **0.045** |
| F | 12 (16%) | 54 (22%) | 32 (13%) | 1 (8.3%) |  |
| M | 64 (84%) | 196 (78%) | 222 (87%) | 11 (92%) |  |
| Weight (kg) | 79 (14) | 78 (15) | 82 (16) | 75 (8) | **0.020** |
| Height (cm) | 171 (7) | 170 (8) | 170 (7) | 167 (6) | 0.3 |
| ICD | 34 (45%) | 128 (51%) | 134 (53%) | 7 (58%) | 0.6 |
| CRT | 18 (24%) | 83 (33%) | 59 (23%) | 4 (33%) | 0.068 |
| Diabetes Mellitus | 34 (45%) | 83 (34%) | 98 (39%) | 2 (17%) | 0.12 |
| Hypertension | 65 (87%) | 171 (69%) | 221 (87%) | 10 (83%) | **<0.001** |
| History of Atrial Fibrillation | 30 (39%) | 85 (34%) | 79 (31%) | 10 (83%) | **0.002** |
| Non-ischaemic Dilated Cardiomyopathy | 24 (32%) | 114 (46%) | 80 (31%) | 4 (33%) | **0.005** |
| Ischaemic Heart Disease | 47 (63%) | 129 (52%) | 168 (66%) | 8 (67%) | **0.014** |
| Baseline NYHA Class |  |  |  |  | **0.005** |
| 1 | 4 (5.3%) | 17 (6.8%) | 12 (4.7%) | 0 (0%) |  |
| 2 | 41 (54%) | 143 (57%) | 151 (59%) | 12 (100%) |  |
| 3 | 29 (38%) | 90 (36%) | 91 (36%) | 0 (0%) |  |
| 4 | 2 (2.6%) | 0 (0%) | 0 (0%) | 0 (0%) |  |
| 12-Month NYHA Class |  |  |  |  | 0.2 |
| 1 | 12 (20%) | 52 (21%) | 43 (17%) | 1 (8.3%) |  |
| 2 | 37 (61%) | 147 (60%) | 176 (69%) | 11 (92%) |  |
| 3 | 12 (20%) | 43 (18%) | 35 (14%) | 0 (0%) |  |
| 4 | 0 (0%) | 2 (0.8%) | 0 (0%) | 0 (0%) |  |
| Last follow-up NYHA Class |  |  |  |  | 0.2 |
| 1 | 15 (28%) | 78 (32%) | 66 (26%) | 4 (33%) |  |
| 2 | 30 (56%) | 132 (54%) | 147 (58%) | 8 (67%) |  |
| 3 | 7 (13%) | 36 (15%) | 39 (15%) | 0 (0%) |  |
| 4 | 2 (3.7%) | 0 (0%) | 2 (0.8%) | 0 (0%) |  |
| Baseline SBP (mmHg) | 124 (13) | 123 (15) | 129 (16) | 132 (23) | **<0.001** |
| 12-Month SBP (mmHg) | 118 (17) | 117 (14) | 122 (16) | 138 (33) | **<0.001** |
| Last follow-up SBP (mmHg) | 118 (15) | 117 (15) | 119 (15) | 126 (13) | 0.2 |
| Baseline DBP (mmHg) | 74 (10) | 73 (10) | 73 (10) | 73 (11) | >0.9 |
| 12-Month DBP (mmHg) | 68 (11) | 69 (10) | 70 (9) | 75 (9) | 0.3 |
| Last follow-up DBP (mmHg) | 70 (11) | 70 (9) | 69 (10) | 70 (0) | >0.9 |
| Baseline Creatinine (mg/dL) | 1.23 (0.41) | 1.15 (0.36) | 1.14 (0.37) | 1.17 (0.14) | 0.4 |
| 12-Month Creatinine (mg/dL) | 1.43 (0.84) | 1.16 (0.39) | 1.15 (0.37) | 1.03 (0.22) | 0.3 |
| Last follow-up Creatinine (mg/dL) | 2.32 (2.40) | 1.33 (0.51) | 1.29 (0.45) | 1.45 (0.39) | **0.031** |
| Baseline K (mEq/L) | 4.41 (0.49) | 4.26 (0.46) | 4.28 (0.46) | 4.35 (0.55) | 0.3 |
| 12-Month K (mEq/L) | 4.43 (0.36) | 4.36 (0.39) | 4.37 (0.44) | 4.50 (0.31) | 0.4 |
| Last follow-up K (mEq/L) | 4.67 (0.58) | 4.47 (0.46) | 4.47 (0.50) | 4.16 (0.37) | **0.026** |
| Baseline Hb (g/dL) | 13.32 (1.72) | 13.32 (1.63) | 13.44 (1.66) | 14.22 (1.30) | 0.4 |
| 12-Month Hemoglobin (g/dL) | 13.25 (1.83) | 13.17 (1.59) | 13.43 (1.81) | 15.40 (1.39) | **0.084** |
| Last follow-up Hemoglobin (g/dL) | 13.94 (1.98) | 13.57 (1.73) | 13.54 (2.01) | 11.77 (3.72) | 0.5 |
| Baseline NTproBNP (pg/mL) | 1,500 (800 - 3,754) | 1,183 (580 - 2,978) | 698 (327 - 1,689) | 1,200 (473 - 1,980) | **<0.001** |
| 12-Month NTProBNP (pg/mL) | 1,121 (725 - 2,554) | 800 (438 - 1,961) | 520 (227 - 960) | 417 (200 - 2,287) | **<0.001** |
| Last follow-up NTProBNP (pg/mL) | 1,406 (217 - 3,735) | 780 (333 - 1,851) | 520 (144 - 1,300) | 3,285 (432 - 5,950) | **0.003** |
| Baseline LVEDV (mL) | 161 (55) | 180 (59) | 173 (57) | 177 (61) | 0.050 |
| Baseline LVESV (mL) | 111 (52) | 126 (50) | 122 (50) | 119 (53) | **0.079** |
| Baseline LVEF (%) | 33 (8) | 31 (7) | 32 (6) | 33 (5) | 0.3 |
| 12-Month LVEDV (mL) | 155 (64) | 175 (61) | 160 (53) | 179 (69) | **0.017** |
| 12-Month LVESV (mL) | 97 (48) | 117 (50) | 105 (42) | 124 (59) | **0.012** |
| 12-Month LVEF (%) | 37 (7) | 34 (8) | 37 (8) | 34 (8) | **0.003** |
| Last follow-up LVEDV (mL) | 148 (67) | 163 (65) | 151 (51) | 188 (94) | 0.2 |
| Last follow-up LVESV (mL) | 93 (49) | 108 (54) | 95 (45) | 145 (93) | **0.036** |
| Last follow-up LVEF (%) | 37 (9) | 35 (10) | 38 (8) | 34 (12) | **<0.001** |
| Baseline E/e’ | 13.2 (6.3) | 13.7 (6.7) | 13.5 (5.0) | 11.9 (7.7) | 0.4 |
| 12-Month E/e’ | 11.8 (5.3) | 12.5 (5.5) | 11.6 (3.8) | 11.2 (2.6) | 0.9 |
| Last follow-up E/e’ | 11.6 (5.8) | 11.7 (5.2) | 10.9 (3.6) | 10.7 (3.6) | >0.9 |
| Baseline S' mean (cm/s) | 6.48 (2.59) | 7.01 (2.68) | 6.40 (2.00) | 9.00 (0.00) | 0.2 |
| 12-Month S' mean (cm/s) | 6.93 (2.89) | 7.20 (2.61) | 6.78 (2.19) | 9.00 (0.00) | 0.4 |
| Last follow-up S' mean (cm/s) | 7.77 (3.39) | 7.51 (3.25) | 7.01 (2.57) | 11.00 (0.00) | 0.2 |
| Baseline TAPSE (mm) | 19.1 (4.0) | 19.4 (4.2) | 19.2 (4.0) | 18.9 (6.4) | 0.9 |
| 12-Month TAPSE (mm) | 19.4 (4.1) | 19.4 (3.7) | 20.0 (3.9) | 18.9 (4.4) | 0.3 |
| Last follow-up TAPSE (mm) | 20.5 (4.6) | 20.3 (4.3) | 19.8 (4.2) | 19.1 (5.1) | 0.8 |
| Baseline S' tricuspid (cm/s) | 10.49 (2.20) | 10.25 (2.08) | 10.54 (2.50) | 9.50 (2.75) | 0.5 |
| 12-Month S' tricuspid (cm/s) | 11.06 (2.27) | 10.74 (2.22) | 11.14 (2.86) | 12.00 (1.53) | 0.2 |
| Last follow-up S' tricuspid (cm/s) | 12.02 (2.56) | 11.22 (2.37) | 11.22 (2.71) | 12.40 (2.95) | 0.053 |
| Baseline sPAP (mmHg) | 35 (12) | 35 (11) | 33 (9) | 31 (18) | 0.3 |
| 12-Month sPAP (mmHg) | 36 (13) | 34 (9) | 31 (8) | 33 (12) | **0.008** |
| Last follow-up sPAP (mmHg) | 32 (11) | 33 (10) | 30 (8) | 30 (8) | **0.004** |
| Baseline LAV (mL) | 58 (25) | 66 (32) | 73 (30) | 64 (27) | **0.010** |
| 12-Month LAV (mL) | 63 (35) | 63 (31) | 70 (29) | 62 (29) | 0.059 |
| Last follow-up LAV (mL) | 64 (33) | 60 (30) | 68 (29) | 67 (23) | **0.039** |
| Baseline GLS (%) | -12.12 (3.01) | -11.11 (3.17) | -10.12 (3.11) | -10.46 (1.51) | **<0.001** |
| 12-Month GLS (%) | -12.79 (2.54) | -12.20 (3.11) | -12.29 (3.20) | -11.50 (1.93) | 0.6 |
| Last follow-up GLS (%) | -14.13 (2.74) | -13.64 (3.02) | -14.39 (2.75) | -13.60 (0.00) | 0.2 |
| Baseline 6MWT (m) | 289 (63) | 290 (85) | 270 (77) | 330 (0) | 0.2 |
| 12-Month 6MWT (m) | 336 (80) | 330 (88) | 348 (58) | 380 (0) | 0.6 |
| Last follow-up 6MWT (m) | 357 (73) | 355 (88) | 363 (67) | 415 (0) | 0.6 |
| Baseline KCCQ score | 53 (15) | 54 (15) | 50 (14) | 72 (0) | **0.020** |
| 12-Month KCCQ score | 58 (16) | 61 (15) | 66 (11) | 76 (0) | **0.006** |
| Last follow-up KCCQ score | 64 (17) | 65 (16) | 68 (12) | 84 (0) | 0.2 |
| Baseline Beta-Blocker Therapy | 53 (84%) | 195 (90%) | 183 (94%) | 12 (100%) | 0.070 |
| 12-Month Beta-Blocker Therapy | 45 (73%) | 197 (91%) | 185 (95%) | 12 (100%) | **<0.001** |
| Last follow-up Beta-Blocker Therapy | 34 (62%) | 197 (92%) | 187 (96%) | 12 (100%) | **<0.001** |
| Baseline MRA Therapy | 38 (60%) | 166 (77%) | 121 (63%) | 11 (92%) | **0.001** |
| 12-Month MRA Therapy | 32 (63%) | 177 (83%) | 132 (68%) | 9 (75%) | **<0.001** |
| Last follow-up MRA Therapy | 27 (68%) | 171 (81%) | 147 (75%) | 9 (75%) | 0.2 |
| Baseline Digoxin Therapy | 6 (9.5%) | 21 (9.7%) | 23 (12%) | 2 (17%) | 0.8 |
| 12-Month Digoxin Therapy | 2 (3.9%) | 23 (11%) | 23 (12%) | 1 (8.3%) | 0.4 |
| Last follow-up Digoxin Therapy | 3 (7.5%) | 29 (14%) | 21 (11%) | 1 (8.3%) | 0.6 |
| Death at follow-up | 7 (9.2%) | 17 (6.8%) | 10 (3.9%) | 2 (17%) | 0.12 |
| HF Hospitalization at follow-up | 22 (29%) | 59 (24%) | 47 (19%) | 6 (50%) | **0.024** |
| New onset AF at follow-up | 19 (25%) | 57 (23%) | 52 (20%) | 5 (42%) | 0.3 |

**Supplementary Table 3.** *Sensitivity multivariable Cox model for MACE including baseline severity markers and sacubitril/valsartan discontinuation.*

| **Characteristic** | **HR** | **95% CI** | **p-value** |
| --- | --- | --- | --- |
| Age (years) | 1.02 | 1.00, 1.04 | **0.016** |
| History of AF | 3.40 | 2.48, 4.66 | **<0.001** |
| Baseline SBP (mmHg) | 1.00 | 0.99, 1.01 | 0.96 |
| Log Baseline NTproBNP (pg/mL) | 0.93 | 0.81, 1.06 | 0.29 |
| Baseline NYHA Class | 0.85 | 0.66, 1.10 | 0.23 |
| **S/V Discontinuation** | **1.60** | **1.04, 2.48** | **0.034** |

Baseline NT-proBNP was log-transformed. HRs are per 1-unit increase in the corresponding covariate

**Supplementary Table 4:** Univariate and Multivariate Cox Regression Analysis for all cause death

|  | **Univariate** | | | **Multivariate** | | |
| --- | --- | --- | --- | --- | --- | --- |
| **Characteristic** | **HR** | **95% CI** | **p-value** | **HR** | **95% CI** | **p-value** |
| Age (years) | 1.09 | 1.05, 1.14 | <0.001 | 1.18 | 1.09, 1.28 | <0.001 |
| Baseline GLS (%) | 1.00 | 0.87, 1.13 | 0.96 |  |  |  |
| Change in GLS | 0.56 | 0.42, 0.75 | <0.001 | 0.38 | 0.25, 0.58 | <0.001 |
| Baseline LVEF (%) | 0.99 | 0.95, 1.04 | 0.79 |  |  |  |
| Change in LVEF | 0.96 | 0.91, 1.01 | 0.15 | 0.96 | 0.85, 1.08 | 0.50 |
| Baseline 6MWT (m) | 1.00 | 0.99, 1.01 | 0.89 |  |  |  |
| Baseline KCCQ Score | 1.00 | 0.97, 1.04 | 0.92 |  |  |  |
| Change in KCCQ Score | 0.97 | 0.92, 1.01 | 0.12 | 0.98 | 0.92, 1.05 | 0.59 |
| Baseline TAPSE (mm) | 0.87 | 0.80, 0.95 | 0.001 |  |  |  |
| Baseline LAV (mL) | 1.01 | 1.00, 1.02 | 0.17 |  |  |  |
| Baseline Creatinine (mg/dL) | 2.51 | 1.27, 4.96 | 0.008 |  |  |  |
| Baseline NTproBNP (pg/mL) | 1.00 | 1.00, 1.00 | <0.001 |  |  |  |
| Log Change in NTProBNP | 1.04 | 0.66, 1.65 | 0.86 | 1.42 | 0.62, 3.28 | 0.41 |
| S/V Discontinuation | 2.43 | 1.06, 5.60 | 0.03 | 1.37 | 0.56, 3.37 | 0.49 |

**Supplementary Table 5:** Univariate and Multivariate Cox Regression Analysis for HF rehospitalization

|  | **Univariate** | | | | **Multivariate** | | | |
| --- | --- | --- | --- | --- | --- | --- | --- | --- |
| **Characteristic** | | **HR**^1^ | **95% CI**^1^ | **p-value** | **HR**^1^ | **95% CI**^1^ | **p-value** |  |
| Age (years) | | 1.02 | 1.01, 1.04 | 0.008 | 1.02 | 0.98, 1.06 | 0.27 |  |
| Baseline GLS (%) | | 1.00 | 0.93, 1.07 | 0.92 |  |  |  |  |
| Change in GLS | | 0.75 | 0.63, 0.88 | <0.001 | 0.74 | 0.59, 0.94 | 0.012 |  |
| Baseline LVEF (%) | | 0.99 | 0.96, 1.01 | 0.40 |  |  |  |  |
| Change in LVEF | | 0.95 | 0.92, 0.98 | <0.001 | 1.00 | 0.93, 1.07 | 0.93 |  |
| Baseline 6MWT (m) | | 1.00 | 1.00, 1.01 | 0.15 |  |  |  |  |
| Baseline KCCQ Score | | 1.03 | 1.01, 1.05 | <0.001 |  |  |  |  |
| Change in KCCQ Score | | 0.94 | 0.92, 0.96 | <0.001 | 0.93 | 0.90, 0.96 | <0.001 |  |
| Baseline TAPSE (mm) | | 0.97 | 0.93, 1.01 | 0.14 |  |  |  |  |
| Baseline LAV (mL) | | 1.00 | 0.99, 1.01 | 0.79 |  |  |  |  |
| Baseline Creatinine (mg/dL) | | 1.63 | 0.96, 2.75 | 0.069 |  |  |  |  |
| Baseline NTproBNP (pg/mL) | | 1.00 | 1.00, 1.00 | 0.072 |  |  |  |  |
| Log Change in NTProBNP | | 1.60 | 1.21, 2.11 | 0.001 | 1.25 | 0.79, 2.00 | 0.34 |  |
| S/V Discontinuation | | 1.89 | 1.19, 2.99 | 0.007 | 0.86 | 0.38, 1.93 | 0.71 |  |

**Supplementary Table 6:** Univariate and Multivariate Cox Regression Analysis for new-onset atrial fibrillation

|  | **Univariate** | | | **Multivariate** | | |
| --- | --- | --- | --- | --- | --- | --- |
| **Characteristic** | **HR**^1^ | **95% CI**^1^ | **p-value** | **HR**^1^ | **95% CI**^1^ | **p-value** |
| Age (years) | 1.03 | 1.01, 1.05 | <0.001 | 1.02 | 0.99, 1.06 | 0.17 |
| Baseline GLS (%) | 0.86 | 0.81, 0.93 | <0.001 |  |  |  |
| Change in GLS | 0.66 | 0.56, 0.78 | <0.001 | 0.65 | 0.53, 0.81 | <0.001 |
| Baseline LVEF (%) | 1.03 | 1.01, 1.06 | 0.016 |  |  |  |
| Change in LVEF | 0.99 | 0.97, 1.02 | 0.63 | 1.00 | 0.94, 1.05 | 0.91 |
| Baseline 6MWT (m) | 1.00 | 1.00, 1.00 | 0.44 |  |  |  |
| Baseline KCCQ Score | 1.03 | 1.01, 1.05 | <0.001 |  |  |  |
| Change in KCCQ Score | 0.93 | 0.91, 0.95 | <0.001 | 0.93 | 0.90, 0.96 | <0.001 |
| Baseline TAPSE (mm) | 1.02 | 0.98, 1.07 | 0.26 |  |  |  |
| Baseline LAV (mL) | 1.00 | 0.99, 1.00 | 0.46 |  |  |  |
| Baseline Creatinine (mg/dL) | 0.87 | 0.46, 1.64 | 0.66 |  |  |  |
| Baseline NTproBNP (pg/mL) | 1.00 | 1.00, 1.00 | 0.98 |  |  |  |
| Log Change in NTProBNP | 1.61 | 1.24, 2.10 | <0.001 | 1.57 | 1.00, 2.45 | 0.05 |
| S/V Discontinuation | 1.61 | 0.99, 2.62 | 0.055 | 1.58 | 0.99, 2.54 | 0.057 |

**Supplementary Table 7.** Availability and missingness of key variables at baseline, 12 months, and last follow-up.

| **Variable** | **Baseline missing n (%)** | **12 months missing n (%)** | **Last follow-up missing n (%)** |
| --- | --- | --- | --- |
| BMI | 48 (8.1%) | — | — |
| BSA | 48 (8.1%) | — | — |
| NYHA class | 0 (0.0%) | 21 (3.5%) | 26 (4.4%) |
| SBP | 16 (2.7%) | 42 (7.1%) | 51 (8.6%) |
| DBP | 16 (2.7%) | 42 (7.1%) | 51 (8.6%) |
| Sodium (Na) | 20 (3.4%) | 48 (8.1%) | 85 (14.4%) |
| Creatinine | 33 (5.6%) | 38 (6.4%) | 29 (4.9%) |
| NT-proBNP | 29 (4.9%) | 10 (1.7%) | 21 (3.5%) |
| KCCQ | 45 (7.6%) | 56 (9.5%) | 49 (8.3%) |
| 6-minute walk test (6MWT) | 47 (7.9%) | 65 (11.0%) | 69 (11.7%) |
| LVEF | 0 (0.0%) | 11 (1.9%) | 8 (1.4%) |
| GLS | 33 (5.6%) | 55 (9.3%) | 47 (7.9%) |
| LAVi | 23 (3.9%) | 44 (7.4%) | 37 (6.2%) |
| E/e′ | 57 (9.6%) | 45 (7.6%) | 61 (10.3%) |
| TAPSE | 50 (8.4%) | 55 (9.3%) | 49 (8.3%) |
| sPAP | 54 (9.1%) | 43 (7.3%) | 61 (10.3%) |

**Supplemental Figure 1**


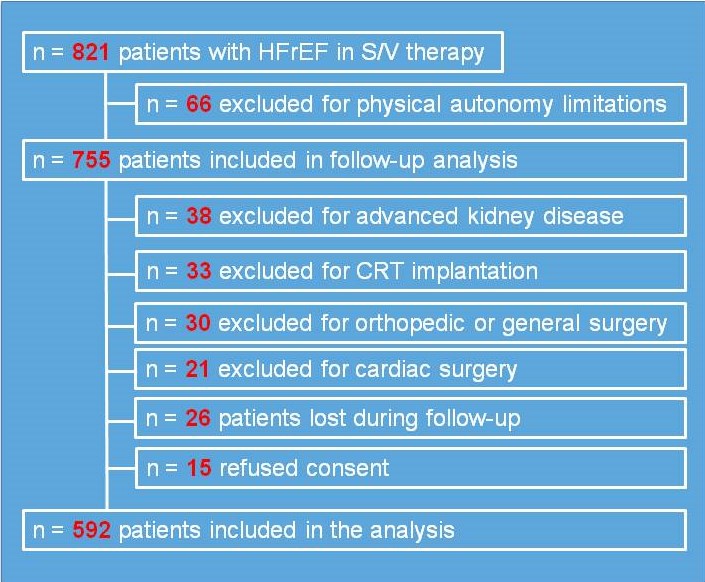

Supplement: xvag082_Supplementary_Data [file xvag082_supplementary_data.docx]
